# AP-1 as a Regulator of MMP-13 in the Stromal Cell of Giant Cell Tumor of Bone

**DOI:** 10.1155/2011/164197

**Published:** 2011-02-27

**Authors:** Isabella W. Y. Mak, Robert E. Turcotte, Snezana Popovic, Gurmit Singh, Michelle Ghert

**Affiliations:** ^1^Department of Surgery, McMaster University, Hamilton, ON, Canada L8S 4L8; ^2^Department of Surgery, Juravinski Cancer Centre, Hamilton Health Sciences, Hamilton, ON, Canada L8V 5C2; ^3^Department of Orthopaedic, McGill University Health Centre, Montreal General Hospital, QC, Canada H3G 1A4; ^4^Department of Pathology and Molecular Medicine, McMaster University, Hamilton, ON, Canada L8S 4L8

## Abstract

Matrix-metalloproteinase-13 (MMP-13) has been shown to be an important protease in inflammatory and neoplastic conditions of the skeletal system. In particular, the stromal cells of giant cell tumor of bone (GCT) express very high levels of MMP-13 in response to the cytokine-rich environment of the tumor. We have previously shown that MMP-13 expression in these cells is regulated, at least in part, by the RUNX2 transcription factor. In the current study, we identify the expression of the c-Fos and c-Jun elements of the AP-1 transcription factor in these cells by protein screening assays and real-time PCR. We then used siRNA gene knockdown to determine that these elements, in particular c-Jun, are upstream regulators of MMP-13 expression and activity in GCT stromal cells. We conclude that there was no synergy found between RUNX2 and AP-1 in the regulation of the MMP13 expression and that these transcription factors may be independently regulated in these cells.

## 1. Introduction

Bone resorption involves dissolution of the nonorganic component of bone, specifically hydroxyapatite, followed by degradation of the collagenous component composed mostly of type-I collagen [1]. Although osteoclasts and their ability to create a highly acidic environment are considered necessary for the degradation of hydroxyapatite, the organic components of bone can be degraded at a physiologic pH with varying efficiencies by the type-I collagenases (Matrix metalloproteinases- (MMPs-) 1, -8 and -13) and the gelatinases (MMP-2 and -9) [2, 3].

Giant cell tumor of bone (GCT) is an aggressive and highly osteolytic bone tumor that is characterized by rapid bone destruction. The cellular elements of GCT include both osteoclast-like giant cells and osteoblast-like stromal cells [4]. Previous work in our lab has shown that the stromal cells respond to the cytokine-rich environment of the tumor with altered expression of MMPs, predominantly with very high levels of MMP-13 [5]. We have also shown that stromal cell-expressed MMP-13 is, at least, partially responsible for optimizing the bone resorption capabilities of the giant cells, likely by recruiting them to the bone surface [6]. Finally, we have shown that the expression of MMP-13 in the stromal cells is regulated, in part, by the Runx2 transcription factor [7].

The Runx2 binding site OSE2 has been found to colocalize with the activator protein 1 (AP-1) transcription factor binding site (not limited to the c-Fos and c-Jun elements) in the promoter region of MMP-13 in human cells [8]. The objective of this study was to use GCT stromal cells as a model for determining the functional regulation of MMP-13 via AP-1 in the human bone environment in order to further our understanding of the physiology of metalloproteinase-induced osteolysis.

## 2. Materials and Methods

### 2.1. GCT Sample Collection

The use of all patient-derived material was approved by our institution's Research Ethics Board, and patient informed consent was obtained individually. The diagnosis of GCT of bone was established by biopsy prior to surgical excision. Specimens were obtained at the time of surgery from patients undergoing tumor resection, and a bone pathologist verified the diagnosis of GCT postoperatively. Tissue samples from four cases of GCT of bone were used in this study, and all experiments were performed in triplicate or as otherwise stated for all four bone tumors.

### 2.2. Primary Cell Lines and Cultures

We established primary cell cultures of GCT stromal tumor cells from fresh GCT tissue. The specimens were freshly minced in Dulbecco's Modified Eagle Medium (D-MEM, Gibco, Burlington, ON) producing a cell suspension with small fragments of tissue. The resultant suspension was passed through a 20-gauge needle prior to seeding in cell culture flasks with D-MEM supplemented with 10% fetal bovine serum (FBS), 2 mM L-glutamine, 100 U/mL penicillin, and 100 *μ*g/mL streptomycin (Gibco). The cell suspension, together with macerated tissue, was cultured in 37°C humidified air with 5% CO_2_. Culture medium was changed every two to three days until *∼*80% confluence. Confluent cells (*∼*80%) were subcultured after dissociating with trypsin and ethylenediaminetetraacetic acid (EDTA). Following several successive passages, the mesenchymal stromal cells became the predominant cell type whereas the multinucleated giant cells were eliminated from the culture. Primary cultures of the proliferating homogenous stromal tumor cell population obtained after the fifth or sixth passage (without any hematopoietic markers) and up to the tenth passage were used for experiments. Human fetal osteoblast (hFOB) 1.19 cells (American Type Culture Collection, ATCC#CRL-11372) were used as a control cell line. Similarly, hFOB cells were maintained in supplemented D-MEM as described for the GCT cells in optimized conditions. To evaluate the effects of cytokine stimulation (interleukin- (IL-) 1*β*) in the recreated tumor environment as described in our previous study [7], the same number of cells in each GCT fraction were seeded accordingly and treated in serum-free medium with or without 1.0 ng/mL of IL-1*β* (R&D Systems). Cells in all experiments in this study were induced by IL-1*β*.

### 2.3. AP-1 Protein Screening Assay

GCT stromal cells were grown to *∼*80% confluence following the treatments as described above. Cell lysates and serum-free D-MEM conditioned media were collected separately following 24-hour stromal cell culture. Additionally, the total number of cells present at the time of the conditioned medium collection was determined by hemocytometer. Cytoplasmic and nuclear cellular fractionation was performed as follows: cells were washed with cold PBS twice followed by the addition of ice-cold cytoplasmic lysis buffer (10 mM Tris pH 7.6, 10 mM KCl, 5 mM MgCl2, 0.2% NP40, protease inhibitor cocktail (Roche, QC, Canada), 0.5 mM PMSF, 1 mM DTT, and 10 *μ*g/mL leupeptin). The cells were scraped and incubated on ice for 10 min. Cellular extracts were acquired by centrifuging the cell suspension at 3500 rpm for 5 min at 4°C. The resulting cytoplasmic supernatant was transferred to a clean tube and the nuclear pellet was washed with lysis buffer and centrifuged at 3500 rpm for 5 min at 4°C. The nuclear pellet was resuspended in ice-cold nuclear extraction buffer for 30–60 min at 4°C and then centrifuged at 16000 rpm for 5 min. Total protein content in the lysates was quantified using the Bradford assay procedure (Pierce Biotechnulogy, IL). AP-1 activity was determined using AP-1 EZ-TFA Transcription Factor Assay (Millipore, MA) according to manufacturer's protocol. Briefly, the nuclear extracts in the supernatant (5 *μ*g/5 *μ*L) from each sample were incubated in 96-well plates coated with a double-stranded biotinylated oligonucleotide containing the flanked DNA binding consensus sequence for the AP-1 family (5′-TGA(C/G)TCA-3′) for 1 hour, then with specific primary AP-1 antibody for 1 hour, and with peroxidase-conjugated secondary antibody (1 : 500) for 1 hour, and subsequently with peroxidase-conjugated secondary antibody (1 : 1000) for 30 min at room temperature. After the colorimetric reaction, optical density was read at 450 nm. For competition assays, cell extracts were incubated with AP-1 specific competitor oligonucleotide (5′-TGA(C/G)TCA-3′).

### 2.4. siRNA Transfection

Mesenchymal stromal cells of GCT were trypsinized and transfected with Runx2, c-Jun, and c-Fos small interfering RNAs (siRNAs) via electroporation. Stromal cells of GCT were washed and resuspended in Opti-MEM I reduced-serum medium (Gibco). Subsequently, cell suspension was mixed with either 200 nM of ON-TARGETplus SMARTpool Runx2 siRNA (Thermo Scientific-Dharmacon), Stealth c-Fos and c-Jun siRNA (Invitrogen), a positive Silencer siRNA control against glyceraldehyde-3-phosphate dehydrogenase (GAPDH), or a nonspecific negative control no. 1 (Ambion Inc.). Stromal cells with siRNA mixture were electroporated using the Gene Pulser II electroporation apparatus (Bio-Rad Laboratories) under a single-pulse protocol with optimized combinations of voltage and capacitance. Then, cells were plated in cell culture flasks with supplemented D-MEM. At 48 hours after the transfection, cells were harvested for Runx2 mRNA. Ribosomal protein S18 (RPS18) was selected among other housekeeping genes for normalization in real-time PCR analysis since GAPDH has been used as the positive siRNA control. The viability of stromal cells after transfection was evaluated by hemocytometry.

### 2.5. RNA Purification and Reverse Transcription (RT)

Total RNA was isolated from GCT stromal cells using the RNeasy Mini Kit (Qiagen, ON) as optimized in our lab. To ensure complete removal of contaminating genomic DNA prior to first-strand synthesis, RNase-free DNase I treatment was applied on the RNeasy column during total RNA isolation. Single-stranded complementary DNA (cDNA) was synthesized from 1.0 *μ*g of total RNA using the SuperScripts III First-Strand Synthesis System for RT-PCR (Invitrogen) and oligo(dT) 12–18 primer, following the manufacturer's instructions.

### 2.6. PCR and Real-Time PCR

The expression of GAPDH, Runx2, MMP-13, c-Fos, and c-Jun in cells treated with various siRNAs were analyzed using real-time RT-PCR. In brief, real-time PCR analysis was performed on cDNA synthesized from GCT stromal total RNA using the MiniOpticon Real-Time PCR Detection System with the iQ SYBR Green Supermix (Bio-Rad Laboratories, ON) according to the manufacturer's instructions. Cycling consisted of 40 cycles of 15 s at 95°C, 30s at 58°C, and 30 s at 72°C, operated with the Opticon Monitor software v3.1. PCR experiments were performed in triplicate and included negative notemplate controls. Primer pairs (Table 1) that spanned at least one intron-exon boundary and produced amplicons in the range of 100–200 bp, were designed using the Real-time PCR Primer Design software (VWR GenScript Corp., Piscataway, NJ), and synthesized (Sigma-Aldrich, ON). In addition, a primer pair for the housekeeping/reference gene RPS18 was also included. We verified the amplicon specificity and sensitivity of all primer pairs with PCR before applying to real-time PCR. PCR products were also separated by gel electrophoresis using a 8% polyacrylamide gel. Bands were visualized by UV illumination of SybrGold-stained gels and captured using a Molecular Imager Gel Doc XR System (Bio-Rad Laboratories). Band intensity was quantitatively analyzed by Quality One software v4.6 (Bio-Rad Laboratories).

### 2.7. Relative Quantification Using Real-Time PCR

The expression level of RPS18 was stable during siRNA treatments. Therefore, RPS18 was designated as the reference gene for relative quantification, through which the expression of endogenous mRNAs from GCT stromal cells was normalized with. Cycle threshold numbers (Ct) were derived from the exponential phase of PCR amplification. Relative changes in mRNA expression were calculated using the comparative ΔΔCT (crossing point) method.

### 2.8. MMP-13 Enzyme-Linked Immunosorbent (ELISA) Activity Assays

Conditioned media were collected and concentrated using an Amicon Ultra-4 Centrifugal Filter Device (Millipore, Billerica, MA). MMP-13 activity was measured using the SensoLyte Plus 520 MMP-13 Assay Kit (AnaSpec Inc.). Concentrated conditioned media were added into wells of a microtiter plate coated with MMP-13 specific antibodies. After 2-hour incubation at room temperature, APMA was added to each well to activate pro-MMP-13. Then, with the substrate incubation, color development from MMP-13 activity was measured using the plate reader, with an excitation and emission wavelength of 485 ± 20 nm and 530 ± 25 nm, respectively. Concentrated fresh media not exposed to cells were used as a negative control.

### 2.9. Statistical Analysis

GraphPad Prism software (GraphPad Software, Inc., USA) was used for statistical analysis. All data are presented as mean ± standard error of the mean (SEM), and are representative of measurements that were performed on four different GCT patient samples (*n* = 4). To assess variations in real-time PCR gene expression, analysis of variance (ANOVA) and the post hoc multiple comparison Tukey's test (*P* < .05) were applied. Measurements were normalized to the negative control treated with nonspecific random siRNA. Each experiment was performed at least three times. *P* values  <.05 were considered to be statistically significant.

## 3. Results

### 3.1. Expression of AP-1 Protein in the Nucleus of GCT Stromal Cells

**Figure 1:**
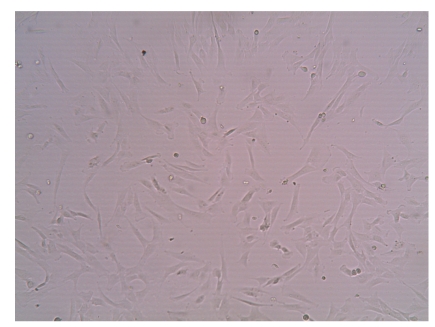
Cell morphology of homogeneous GCT stromal cells induced by IL-1*β*. Representative picture was taken with light microscope at magnification ×400.

**Figure 2:**
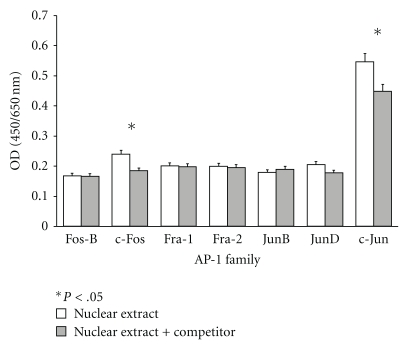
Expression of AP-1 proteins in the nucleus of GCT stromal cells. The AP-1 screening assay examines the protein level of FosB, c-Fos, Fra-1, Fra-2, JunB, JunD, and c-Jun of the AP-1 family in the nuclear extracts of IL-1*β* stimulated stromal cells. The specific competitor was used to determine specific binding. Values represent the means ± SEM of triplicate experiments after being normalized to the background control. *P < .05 is versus corresponding conditions with specific competitor. Statistical comparison by analysis of variance with post hoc Tukey's tests.

To identify which members of the AP-1 family are present in GCT stromal cells, an AP-1 screening assay was used to detect specific transcription factor DNA binding activity in the nuclear extracts. GCT stromal cells stimulated with IL-1*β* were isolated and passaged from primary patient tissue sample into homogeneous mesenchymal stromal cells, as shown in Figure 1, before extracting the lysate. The AP-1 screening assay examines both the Jun (c-Jun, JunB, and JunD) and Fos (c-Fos, FosB, Fra-1, and Fra-2) families in AP-1. The specific competitor double-stranded oligonucleotide was included as an important control for verifying the specificity of the colorimetric signal resulting from protein binding to the labeled AP-1 probe. Only c-Fos and c-Jun were shown to be significantly present in GCT stromal cells (Figure 2). Both were reduced when the specific competitor was added to the lysate, indicating specific binding.

### 3.2. MMP-13 Expression with AP-1 and Runx2 Knockdown

**Figure 3:**
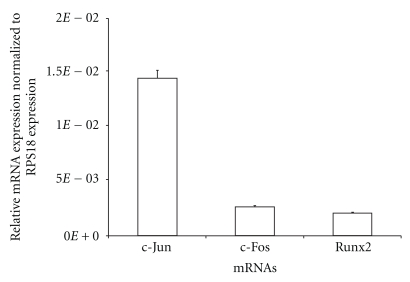
Relative mRNA expression of the Runx2 and AP-1 transcription factors based on real-time RT-PCR. The expression of c-Jun, c-Fos, and Runx2 in GCT stromal cells treated with cytokines IL-1*β* for 24 h in serum-free media was analyzed using real-time PCR. The ΔΔCT method was used to calculate the real-time RT-PCR fold change using RPS18 mRNA for normalization, and all changes in expression are relative to the control without any treatment. Triplicate independent real-time PCR were performed.

To re-confirm the presence and expression of c-Fos and c-Jun of AP-1 in GCT stromal cells, the baseline mRNA expression level of c-Fos, c-Jun, and Runx2 was determined using real-time PCR. The mRNA expression of these three transcription factors was relatively low into the hundredth level relative to RPS18 expression (Figure 3). c-Fos had a similar expression level to that of Runx2, but c-Jun exhibited a 5-6-fold higher expression level.

**Figure 4:**
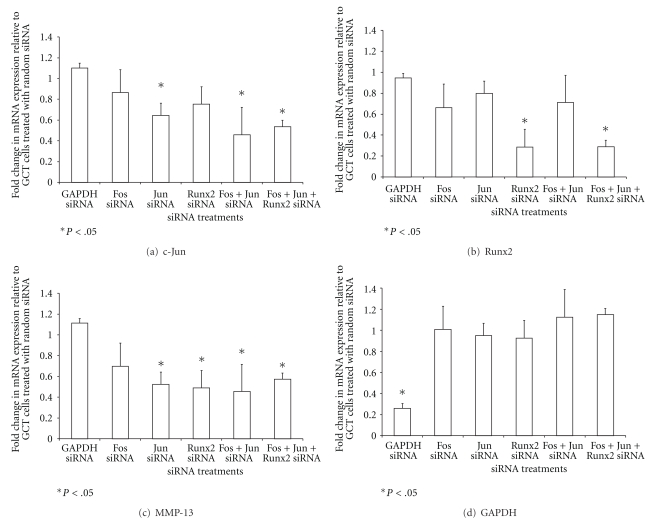
The effect of siRNA knockdown on c-Fos, c-Jun, Runx2, MMP-13, and GAPDH mRNA expression in the IL-1*β* -induced mesenchymal stromal cells of GCT. GCT stromal cells were transfected by electroporation with corresponding siRNA for 48 h. Treated samples were examined using real-time PCR. The ΔΔCT method was used to calculate the real-time RT-PCR fold change using RPS18 mRNA as an endogenous control. Three independent experiments were performed. mRNA expression of (a) c-Jun, (b) Runx2, (c) MMP-13, and (d) GAPDH upon treatment with combinations of siRNA. *P < .05 is versus random nonspecific siRNA control. Statistical comparison by analysis of variance with post hoc Tukey's tests.

Next, to further elucidate the role of AP-1 and Runx2 in MMP-13 transcriptional regulation, we depleted various combinations of c-Fos, c-Jun, and Runx2 in the mesenchymal stromal cells of GCT by using RNA interference. Random siRNA served as the negative control. All siRNA treatments (c-Fos, c-Jun, Runx2, and c-Fos + c-Jun, c-Fos + c-Jun + Runx2) were normalized to the random siRNA negative control. The expression of c-Jun was 40–60% suppressed when treated with c-Jun, and c-Fos + c-Jun, c-Fos + c-Jun + Runx2 siRNA, respectively (Figure 4(a)). More than 70% of Runx2 expression was depleted in both Runx2 and c-Fos + c-Jun + Runx2 siRNA conditions, as detected by real-time PCR (Figure 4(b)). Importantly, MMP-13 expression was decreased by 40–50% following knockdown of c-Jun alone, Runx2 alone, c-Fos and c-Jun in combination, and c-Fos, c-Jun and Runx2 in combination (Figure 4(c)). Yet, the lack of effect in MMP-13 suppression may be due to the low efficiency of the cFos knockdown. As a test of transfection efficiency, GAPDH mRNA was decreased by 70% when treated with the GAPDH siRNA but unaffected in the random and other siRNA conditions (Figure 4(d)).

### 3.3. MMP-13 Activity with AP-1 and Runx2 Knockdown

**Figure 5:**
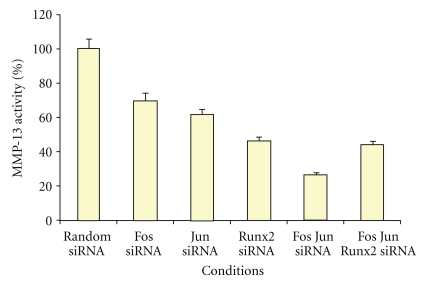
The effect of siRNA knockdown on the level of MMP-13 activity in IL-1*β*-stimulated GCT cells. Filtered and concentrated conditioned media from siRNA silenced GCT stromal cells were analyzed using the MMP-13 activity kit as per optimization in our lab. Results of the ELISA activity assay are shown in triplicate with error bars. The MMP-13 activity levels were normalized to the amount of total protein for each condition.

To validate the effect of silencing c-Fos, c-Jun, and Runx2 on their downstream target MMP-13 at the translational level, MMP-13 enzyme activity was measured from culture medium collected from the siRNA treatments. The percentage of MMP-13 activity (Figure 5) exhibited a similar trend to measurements from the MMP-13 real-time PCR data (Figure 4(c)). However, c-Fos siRNA demonstrated a 30% knockdown of MMP-13 activity as shown in Figure 5, as compared to its insignificant effect on MMP-13 transcription. c-Jun and Runx2 gene silencing resulted in 40% and 55% knockdown in MMP-13 activity, respectively. Interestingly, AP-1 knockdown resulted in greater MMP-13 activity silencing than did Runx2 knockdown, indicating that both transcription factors play a role in MMP-13 expression and activity in these cells.

## 4. Discussion

The regulation of MMPs plays an important role in tissue remodeling associated with various physiological and pathological processes involving turnover of the extracellular matrix. MMP-13 has a key role in the MMP activation cascade and appears to be critical in bone metabolism, homeostasis, osteoarthritis, and rheumatoid arthritis [9], but is also highly associated with tumor invasion and metastasis [10]. A high level of MMP-13 expression is the unique gene profile signature of the stromal cells of GCT compared to other tissues and cell lines [5]. The surge of MMP-13 expression in GCT stromal cells is induced by cytokines secreted by the multinucleated giant cells in the tumor environment [7].

Intervention affecting the regulation of MMPs in pathologic tissues has substantial clinical potential. At least 56 MMP inhibitors have been assessed as candidates in various therapeutic areas, mainly for targeting cancer, arthritis, or cardiovascular diseases [11]. Due to unacceptable side effect profiles, direct MMP inhibitors have failed in many clinical trials [12, 13]. Thus, other targeted treatment alternatives are needed, such as aiming at a higher level of regulation of MMP-13 by transcription factors.

To get a broader scope of how transcription factors regulate the cytokine-stimulated MMP-13 expression in GCT stromal cells, we specifically examined AP-1 in this study to extend our understanding of Runx2 as a modulator of MMP-13. Both Runx2 and AP-1 have binding sites in the promoter region of MMP-13. Selvamurugan et al. reported that activation of the MMP-13 promoter requires both the AP-1 and Runx2 sites *in vitro* and *in vivo* conditions in mice [14], as well as in cultured osteosarcoma cell lines [15]. 

AP-1 is a transcription factor, which is a heterodimeric protein, composed of proteins belonging to the c-Fos, c-Jun, ATF, and JDP families. It regulates gene expression in response to a variety of stimuli, including cytokines, growth factors, stress, bacterial, and viral infections [16]. AP-1 in turn controls a number of cellular processes including differentiation, proliferation, and apoptosis [17]. Since AP-1 consists of multiple protein families, two central members, c-Fos and c-Jun, were identified as highly expressed in GCT stromal cells using both the AP-1 family screening assay and real-time PCR. 

The results of siRNA knockdown in GCT cells showed that silencing c-Jun and Runx2 significantly silenced the expression of MMP-13. However, knocking down all three transcription factors in the c-Fos + c-Jun + Runx2 siRNA condition did not reduce the MMP-13 expression further, indicating a baseline endogenous expression level of MMP-13 controlled by other factors or pathways. This result also suggests that no synergistic interaction happened between the AP-1 and Runx2 transcription factors in MMP-13 regulation. We found the c-Jun element of AP-1 to show higher expression and greater control over MMP-13 expression in GCT stromal cells that the c-Fos element of AP-1. Both c-Fos and c-Jun are end targets of the ERK and JNK pathways such that inhibiting either pathway in our previous study demonstrated a similar trend in subduing MMP-13 expression [7]. Therefore, upstream signaling may play a key role in recruiting the elements of AP-1 in MMP-13 regulation. 

From the results of this study, it is clear that the functional regulation of MMP-13 requires both the AP-1 and Runx2 transcription factors in our GCT stromal cell model. Complex regulatory loops exist in the cells of the skeletal system, which spatially and temporally control the progression of bone and cartilage cell maturation and coordinate it with events in surrounding tissues [18]. Transcription factors are downstream in the signaling cascade where cells are exposed to extracellular or intercellular signals. What controls these transcription factors could be the ultimate target for clinical intervention in bone pathology. For example, it has been shown that parathyroid hormone-related peptide (PTHrP) delays chondrocyte differentiation by suppressing Runx2 through a feedback regulatory loop in which Indian hedgehog (Ihh) induces PTHrP expression, while PTHrP in turn downregulates Ihh [19–21]. Hence, examining the relationship between PTHrP, Ihh, Runx2, and MMP-13 in the bone microenvironment and tumorigenesis in GCT would be an interesting followup to the current study.

In summary, we have demonstrated that c-Fos and c-Jun of the AP-1 family are expressed by GCT stromal cells. The cytokine-induced MMP-13 expression in these cells is strongly suppressed by various combinations of c-Fos, c-Jun, and Runx2 gene knockdown. Our results indicate that c-Fos, c-Jun, and Runx2 all regulate MMP-13 expression and activity to a certain degree in GCT mesenchymal stromal cells. We propose that cytokines secreted by multinucleated giant cells stimulate MMP-13 production in the GCT stromal cells through both AP-1 and Runx2 transcription factors. The regulation of these transcription factors may therefore serve as targets in treatment strategies for this destructive tumor and other degenerative diseases of the musculoskeletal system where MMP-13 is the most prominently implicated protease. Nevertheless, more evidence is needed to clarify what upstream extracellular or intercellular signals modulate AP-1 and Runx2 in controlling MMP-13 regulation in the human bone environment in order to further our understanding of the physiology of metalloproteinase-induced osteolysis.

## Figures and Tables

**Table 1:** Human primer sequences specially designed for real-time RT-polymerase chain reaction (PCR) amplification.

Gene	Forward/Reverse	Primer sequence	Accession no.	Size of product (bp)	Melting temperature (°C)
c-Fos	F	5^'^ AGA ATC CGA AGG GAA AGG AA 3^'^	NM_005252	150	63.6
	R	5^'^ CTT CTC CTT CAG CAG GTT GG 3^'^			63.8
c-Jun	F	5^'^ CAG GTG GCA CAG CTT AAA CA 3^'^	NM_002228	80	63.8
	R	5^'^ GTT TGC AAC TGC TGC GTT AG 3^'^			63.5
MMP-13	F	5^'^ CTT CCC AAC CGT ATT GAT GC 3^'^	NM_002427	143	64.1
	R	5^'^ TTT GGA AGA CCC AGT TCA GA 3^'^			62.2
Runx2	F	5^'^ TCT GGC CTT CCA CTC TCA GT 3^'^	NM_004348	142	64.0
	R	5^'^ AAG GTG GCT GGA TAG TGC AT 3^'^			63.4
RPS18	F	5^'^ GAT GGG CGG CGG AAA ATA G 3^'^	NM_022551	165	68.4
	R	5^'^ GCG TGG ATT CTG CAT AAT GGT 3^'^			65.8
GAPDH	F	5^'^ CAT GAG AAG TAT GAC AAC AGC CT 3^'^	NM_002046	113	62.0
	R	5^'^ AGT CCT TCC ACG ATA CCA AAG T 3^'^			62.5
